# The Mia40/CHCHD4 Oxidative Folding System: Redox Regulation and Signaling in the Mitochondrial Intermembrane Space

**DOI:** 10.3390/antiox10040592

**Published:** 2021-04-12

**Authors:** Eleanor Dickson-Murray, Kenza Nedara, Nazanine Modjtahedi, Kostas Tokatlidis

**Affiliations:** 1 Institute of Molecular Cell and Systems Biology, College of Medical, Veterinary and Life Sciences, University of Glasgow, University Avenue, Glasgow G12 8QQ, UK; e.dickson-murray.1@research.gla.ac.uk; 2 CNRS, Gustave Roussy, Aspects Métaboliques et Systémiques de L’oncogénèse pour de Nouvelles Approches Thérapeutiques, Université Paris-Saclay, 94805 Villejuif, France; kenza.nedara@u-psud.fr

**Keywords:** mitochondria, oxidative folding, redox signaling, Mia40, intermembrane space

## Abstract

Mitochondria are critical for several cellular functions as they control metabolism, cell physiology, and cell death. The mitochondrial proteome consists of around 1500 proteins, the vast majority of which (about 99% of them) are encoded by nuclear genes, with only 13 polypeptides in human cells encoded by mitochondrial DNA. Therefore, it is critical for all the mitochondrial proteins that are nuclear-encoded to be targeted precisely and sorted specifically to their site of action inside mitochondria. These processes of targeting and sorting are catalysed by protein translocases that operate in each one of the mitochondrial sub-compartments. The main protein import pathway for the intermembrane space (IMS) recognises proteins that are cysteine-rich, and it is the only import pathway that chemically modifies the imported precursors by introducing disulphide bonds to them. In this manner, the precursors are trapped in the IMS in a folded state. The key component of this pathway is Mia40 (called CHCHD4 in human cells), which itself contains cysteine motifs and is subject to redox regulation. In this review, we detail the basic components of the MIA pathway and the disulphide relay mechanism that underpins the electron transfer reaction along the oxidative folding mechanism. Then, we discuss the key protein modulators of this pathway and how they are interlinked to the small redox-active molecules that critically affect the redox state in the IMS. We present also evidence that the mitochondrial redox processes that are linked to iron–sulfur clusters biogenesis and calcium homeostasis coalesce in the IMS at the MIA machinery. The fact that the MIA machinery and several of its interactors and substrates are linked to a variety of common human diseases connected to mitochondrial dysfunction highlight the potential of redox processes in the IMS as a promising new target for developing new treatments for some of the most complex and devastating human diseases.

## 1. Introduction

Mitochondria are essential organelles that participate in a diverse range of vital cellular functions ranging from energy production, to iron, macromolecule, and lipid biosynthesis. Mitochondria play also a critical role in specific cell death modalities [1,2,3]. Surrounded by the outer mitochondrial membrane (OMM) and inner mitochondrial membrane (IMM), the mitochondrial intermembrane space (IMS) is the smallest sub-compartment of mitochondria. The IMS can be further sub-divided into the boundary IMS and the cristae space; the latter is formed from invaginations of the IMM. The cristae space is separated from the boundary IMS by cristae junctions (CJs) and contact sites where the OMM and IMM physically contact each other [4,5]. The diameter of the IMS (OMM to IMM) is around 60 nm. However, this small compartment is the interface between the mitochondrial matrix and the cytosol, exchanging various proteins, lipids, metabolites, and metal ions, all of which play a role in redox regulation [6]. This “controlled exchange” of the IMS is due to the presence of porins in the OMM, which allow molecules of less than 5 kDa to freely diffuse, facilitating communication between mitochondria and other areas of the cell [7]. Alongside this “buffering” role between the cytosol and the matrix, IMS proteins are involved in the biogenesis, assembly, and stability of respiratory chain complexes, protein folding, lipid homeostasis, and the initiation of cell death through the release of proteins such as cytochrome *c* [3,8].

Mitochondria contain around 1000 different proteins in *Saccharomyces cerevisiae (S. cerevisiae)* and roughly 1400 in humans, yet the yeast and human mitochondrial genome encode for just seven and 13 proteins, respectively [9]. Therefore, 99% of mitochondrial proteins are nuclear encoded and require import into their correct location within the organelle. The main pathways for mitochondrial protein import are the presequence pathway for matrix proteins and the carrier pathway for IMM proteins. These two major pathways both contain steps where the IMS plays a crucial role in maintaining the imported preproteins in a reduced and unfolded state. The IMS itself contains ≈51 proteins in the yeast *S. cerevisiae* [10] and ≈53 in humans [11]. We should note here that a previous estimate for the number of IMS proteins for human mitochondria brought this number to 127 [12]. The difference is due to the fact that Rath et al. (2020) annotated proteins that are part of a membrane complex in their mature form as membrane proteins even though they are IMS-localised during their biogenesis (leading to a potential underestimation), and the APEX2 proximity biotinylation used by Hung et al. (2014) labelled not only IMS proteins but also those in the MOM and MIM that are accessible to the biotin label emanating from the IMS (leading to a potential overestimation). Interestingly, none of the IMS proteins are encoded by the mitochondrial genome and therefore require additional import pathways. There are six main import pathways to the IMS (recently reviewed by Edwards, Gerlich, and Tokatlidis 2020) with the Mia40 oxidative folding pathway (also known as the disulphide relay) being one of the most wellknown. Additionally, it has been shown that a number of IMS proteins exhibit dual localisation between the cytosol and the IMS, reinforcing the communication that occurs between these two locations [7,10].

The MIA (Mitochondrial Import and Assembly) pathway in the IMS is unique as it is the only pathway that involves chemical modification of cysteine residues and therefore is potentially subject to regulation through redox signalling. This potential is due to the sensitivity that the cysteine residues have to redox states due to the presence of anionic sulfur, which is reactive with different oxidising agents, resulting in multiple oxidised species [13]. The IMS provides a redox active space that is more oxidising than the cytosol, and this redox active space allows for the formation of disulphide bonds through oxidative folding. Additionally, the redox environment in the IMS is maintained separately from the cytosol and the matrix [14]. Disulphide bond formation occurs also in the endoplasmic reticulum (ER) of mammalian cells and the periplasm of bacteria where a balance between an oxidative and reductive pathway is needed for optimal regulation of the redox process. However, unlike those compartments, a reducing pathway in the IMS is yet to be fully described [15]. In broader terms, the concept that mitochondria biogenesis is subject to cell signalling pathways is fast gaining momentum, spurred by numerous important studies in the field. Phosphorylation signalling is an important signalling pathway that is linked to mitochondria biogenesis [16] but will not be described in this review. Here, we will focus on the basic players and protein modulators of the MIA pathway with an emphasis on redox regulation. Additionally, we will discuss the small redox molecules in the IMS and their interplay with proteins controlling the IMS-located import machinery.

## 2. The MIA Pathway—Basic Players and Mechanism

**Figure 1:**
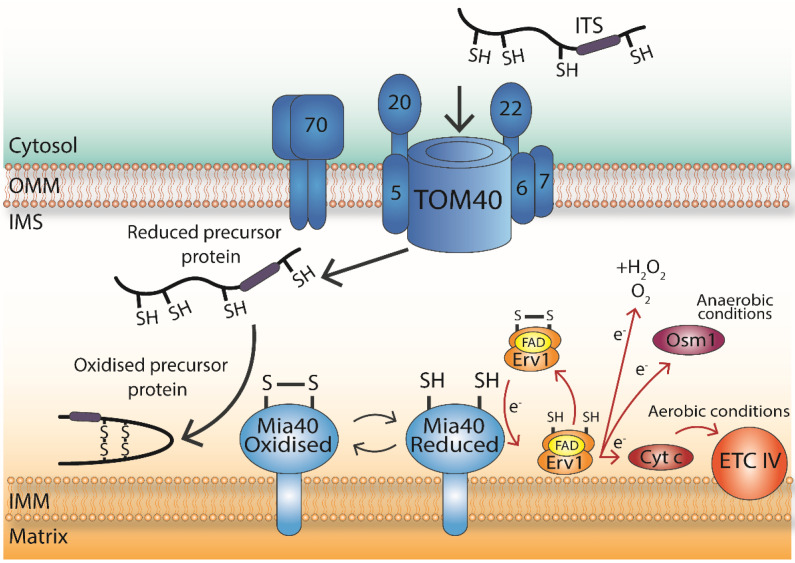
Mechanism and basic players of the MIA pathway. Incoming nuclear-encoded and cysteine-bearing proteins destined for the intermembrane space (IMS)-localised MIA pathway are firstly translocated through the Tranlocase of the Outer Membrane TOM complex. The ITS (IMS-Targeting Signal, shown in purple) interacts with Mia40 through the “sliding–docking” model. Following interaction with Mia40, the substrate is released in an oxidised and stably folded conformation. The Cysteine-Proline-Cysteine CPC motif of Mia40 is left in a reduced state and requires re-oxidation to continue participating in import reactions. The re-oxidation of Mia40 is facilitated by Erv1, and then, the electrons are passed directly to molecular oxygen (resulting in the production of hydrogen peroxide), or to cytochrome c and respiratory chain complex IV in aerobic conditions, or to Osm1 in anaerobic conditions. Overall, the MIA pathway inserts disulphide bonds into proteins, trapping them in the IMS of mitochondria.

The redox active environment of the IMS promotes the oxidation of cysteine residues, which ultimately leads to the formation of disulphide bonds. These disulphide bonds are formed in substrate proteins that are imported into the IMS, in a reaction where the thiol groups of two cysteine residues are oxidised, forming a covalently linked intramolecular disulphide. It is through the process of oxidative folding where a protein acquires its native disulphide bonds and therefore its native three-dimensional structure [17,18]. The MIA pathway in the IMS introduces disulphide bonds to imported proteins through oxidative folding, which allows the retention of the proteins in the IMS in an oxidised and folded state (Figure 1). The pathway is comprised of two essential proteins, the oxidoreductase Mia40 (CHCHD4 in mammals) [17,19] and the flavin adenine dinucleotide FAD-dependent sulfhydryl oxidase Erv1 (ALR, Augmenter of Liver Regeneration, in humans) [20]. This section will focus on the key players and the basic mechanisms of the regulation of this import pathway, which was initially discovered in the yeast model organism.

Mia40 contains six strictly conserved cysteine residues that are organised as a redox-sensitive CPC motif and two CX9C motifs. These two CX9C motifs stabilise the protein and form a hydrophobic cleft of two anti-parallel helices to bind the substrate. The solvent-exposed CPC motif constitutes the redox-active cysteine pair of Mia40 that accepts electrons shuttled from the substrate protein and introduces the disulphide bond whilst switching between a reduced and oxidised state [17,19,21,22].

Classical Mia40 substrates typically have a small mass of between 8 and 22 kDa, a hydrophobic intermembrane space targeting signal (ITS), and either twin CX3C or CX9C motifs, such as the family of Tim proteins [23,24,25]. Other non-typical Mia40 substrates contain different patterns of cysteine residues, for example, the protein Mix23 contains a CX13C/CX14C motif. This indicates that the MIA pathway interacts with a broader range of cysteine motifs [10,26].

To reach the IMS, proteins must pass first through the OMM and, in the MIA pathway protein, substrates are translocated through the TOM complex in an unfolded and reduced conformation which renders them import-competent [18]. During translocation into the IMS (Figure 1), substrate proteins bind to Mia40 via specific docking cysteines, which further drives the import process [27,28]. The mechanism of interaction between Mia40 and the substrate protein involves an IMS-targeting signal (ITS), which is an internal peptide of nine amino acids that forms an amphipathic helix. First described in the protein substrates Tim9 and Tim10, the ITS is sufficient for crossing the OMM and has a complementary fit to the cleft of Mia40 through hydrophobic interactions [25,29]. The mechanism of interaction between Mia40 and the imported protein can be explained by the “sliding–docking” model where the first “sliding” step involves positioning the ITS of the imported protein in the cleft of Mia40 through non-covalent hydrophobic interactions. This initial interaction between the ITS and the hydrophobic cleft, due to the close proximity and correct orientation, leads to the subsequent “docking” step, which involves the formation of a transient intermolecular bond between Mia40 and the substrate, which is mediated by the second cysteine of the CPC motif of Mia40 and the docking cysteine of the ITS signal of the substrate [25,30]. Then, this transient intermolecular disulphide is subjected to nucleophilic attack by the other cysteine of the substrate, creating an intramolecular disulphide within the substrate. Following this, the substrate is released from Mia40 in an oxidised, stably folded conformation, thereby remaining trapped in the IMS. The CPC motif of Mia40 is left in a reduced state and requires re-oxidation to restore its capacity to import substrate proteins [21,25,31].

This is where the second essential component of the MIA pathway Erv1 comes into play and re-oxidises Mia40 for another cycle of disulphide bond formation (Figure 1). Erv1 is a 22 kDa flavin-linked sulfhydryl oxidase that is present in a head-to-tail dimer in the IMS. The basic role of Erv1 in the disulphide relay is to re-oxidise Mia40. Mutated or knockout Erv1 cells have reduced levels of Tims and other small IMS-localised substrate proteins, highlighting the importance of Erv1 in IMS protein import [20]. It has been reported that the evolutionary conserved human homolog of yeast Erv1 (ALR) can complement its yeast counterpart for the function of oxidative biogenesis of mitochondrial IMS proteins [32]. Erv1 family members have a core domain of ≈100 amino acids that are arranged in a four-helix bundle structure with FAD bound non-covalently for stabilisation. The FAD-binding domain contains the stabilising proximal disulphide (C130/133) in the redox active CXXC motif [33,34]. The first pair (C30/33) is the shuttle disulphide, which is located at the N-terminus and interacts with Mia40, whilst the third (C159/176) is the structural disulphide that is recognised by Mia40 during Erv1 import. This N-terminal shuttle domain of Erv1 is sufficient and necessary for the non-covalent interaction of Mia40 and Erv1 [31,35,36,37,38,39].

Oxidised Erv1 interacts with reduced Mia40 following a “substrate mimicry” model where the N-terminal segment of Erv1 (about 72 residues) interacts with the hydrophobic binding groove of Mia40 in a similar manner to the ITS of the substrate, accepting the electrons via its N-terminal redox active CPC motif [38,40]. Then, Erv1 can transfer these electrons either directly to oxygen, which results in the production of hydrogen peroxide in the IMS, or to cytochrome *c* and complex IV of the respiratory chain (Figure 1) [41,42,43,44,45]. Alternative final electron acceptors such as Osm1 in yeast allow the process to proceed under anaerobic conditions [46]. Recent evidence has suggested that Erv1 is perhaps not as active as previously thought and has been shown to be a moderately efficient enzyme, being able to use both O2 and cytochrome *c* as electron acceptors. Such a moderate efficiency could be due to the slower kinetics of protein import and disulphide bond formation, which places no pressure on the requirement for a higher catalytic efficiency [47].

It is worth mentioning that Mia40 requires and interacts with endogenous Mia40 for its own import into the IMS. The import of Mia40 occurs in kinetically distinct steps; firstly, Mia40 translocates through the TOM complex, and it is then inserted into the IMM by the TIM23 complex [48]. Secondly, the core of Mia40 is folded with assistance from endogenous Mia40 through oxidation of the structural CX9C motif. Lastly, there is an interaction with Erv1 to oxidise the CPC motif of Mia40 resulting in a folded and active Mia40 [8,48]. Intriguingly, Erv1 itself is imported via Mia40, but it is not a classical Mia40 substrate, as import is mediated by its CX15C motif found towards the C-terminus of the protein rather than the FAD-proximal CXXC or the N-terminal CXXC shuttle motif [24,37].

Overall, the MIA pathway is responsible for inserting disulphide bonds into proteins, trapping them in the IMS. Similar to the disulphide relay systems in the ER and bacterial periplasm, electrons are transferred from the substrate onto Mia40, then Erv1, and onto a final electron acceptor with Mia40 being re-oxidised in the process (Figure 1). However, unlike the disulphide relay in the ER and bacterial periplasm, the process of disulphide bond insertion in the IMS has no reductive pathway described yet. In 2012, it was reported that in the IMS proteome of yeast mitochondria, the cytosolic reductive system, thioredoxin 1 (Trx1), and its partner thioredoxin reductase 1 (Trr1) are present [10]. This raises questions around the function of these proteins in the IMS and any potential regulatory role, either directly or indirectly, on Mia40 through influencing the redox environment of this sub-compartment.

## 3. The Mia40/CHCHD4 Machinery in Human Cells

Mia40 exists in the IMS as either a soluble protein in higher eukaryotes or anchored through its N-terminal in fungi and yeast (although this membrane anchor is not essential for function). The evolutionary conserved human homolog of yeast Mia40 is the small (16 kDa) IMS-soluble CHCHD4 (Coiled-Coil-Helix-Coiled-Coil-Helix Domain Containing 4) [17,49]. Compared to the membrane-bound Mia40, the CHCHD4 protein has lost its N-terminal moiety that includes the mitochondrial targeting signal (MTS) and the transmembrane domain (TMD), while conserving the C-terminal functional segment that carries the signature CPC and twin CX9C motifs [17,21,26,49,50]. The functional conservation between the two proteins was confirmed by means of RNA interference silencing in human cells and complementation assays demonstrating that CHCHD4 is imported in yeast and is perfectly capable of rescuing the MIA import pathway and cell viability in yeast mutants suppressed for Mia40 [49,51]. While the above-mentioned data were clear indicators of a perfect functional conservation of Mia40/CHCHD4 during the evolution from yeast to man, the absence of the N-terminal segment in the human CHCHD4 protein revealed a major change in the regulation of its biogenesis as well as its capacity to interact with other proteins. Probably one of the most prominent consequences of the loss of the N-terminal MTS and TMD segments is the switch in the molecular mechanisms of the mitochondrial import of the protein. More precisely, contrary to its yeast homolog, CHCHD4 is no longer imported through the activity of the TIM23 translocase in a membrane potential-dependent manner but rather depends on a self-catalytic import mechanism [21,22,49,51,52]. So far, the advantage that this import mechanism shift offers is elusive, but one can speculate that in specialised cells of higher eukaryotes, the redox-regulated import of CHCHD4 would enable an efficient adaptation of the Mia40/CHCHD4 pathway to physiological signals or specific stress conditions. The second noticeable characteristic of CHCHD4 that differentiates it from its yeast homolog is the gain of capacity to interact with the IMM-bound flavoprotein called Apoptosis-Inducing Factor (AIF) [52,53]. The evolutionary advantage of the interaction between CHCHD4 and AIF is not understood, but the data published so far indicate that AIF is needed for the co-translational import of CHCHD4 in the IMS [52]. While the N-terminal 27 residues of CHCHD4 is necessary and sufficient for the physical interaction with AIF [52], the negatively charged C-terminal portion of the protein was found to be critical for fine tuning of the import kinetics and for protection against proteasomal degradation during the import process [54]. In mammals, the AIF-regulated optimal functioning of the CHCHD4/Mia40 import pathway is essential for the biogenesis of the respiratory chain protein complexes [52,53]. In various experimental models, it was shown that the loss of AIF provokes a bioenergetics dysfunction that is mainly caused by CHCHD4 protein deficiency and the subsequent loss of CHCHD4 substrates [26,55]. AIF mutations have been associated with human neurodegenerative mitochondriopathies, and, among these, AIF G308E and dR201del are described for their potential to perturb the AIF/CHCHD4 import pathway [52,53]. The essentiality of the CHCHD4/Mia40 mitochondrial import pathway in higher eukaryotes is reflected by the embryonic lethality, which is caused by the homozygous knockout of *Aif* or *chchd4* genes [55,56].

## 4. Redox Proteins Interacting with the MIA Machinery

As mentioned previously, the MIA pathway is unique, as it is the only import pathway that chemically modifies its substrates, resulting in intramolecular disulphide bonds. This potentially opens up this pathway to regulation by the redox environment of the cell, which is determined by all the oxidative and reductive reactions that occur. Redox reactions produce reactive oxygen species (ROS) and reactive nitrogen species (RNS), which are beneficial for cell signalling at low levels but damaging at high levels. These damaging effects can include DNA modification, protein oxidation, and lipid oxidation, all of which are detrimental to cell function and can lead to cell death [31,57]. The balance between the production and removal of these reactive species has a direct influence on vital cellular processes such as protein biogenesis, which is of particular importance for mitochondria, as ≈99% of mitochondrial proteins require import [15].

**Figure 2:**
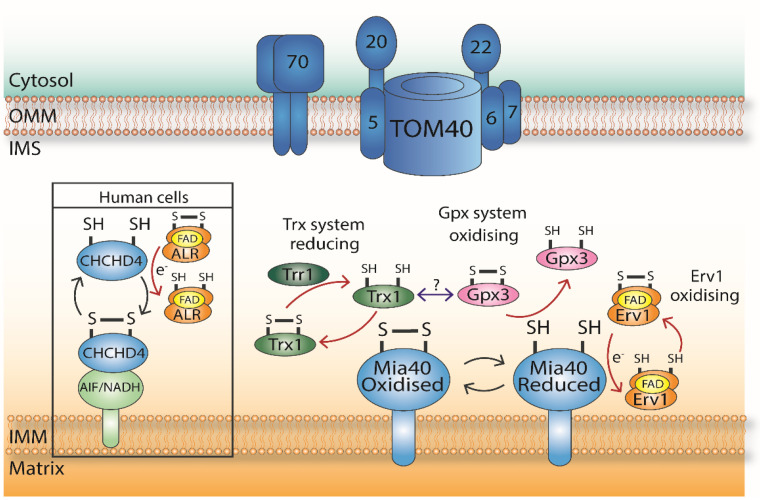
Protein factors modulating Mia40. AIF (Apoptosis-Inducing Factor) controls the import of CHCHD4 (Coiled-Coil-Helix-Coiled-Coil-Helix Domain Containing 4) in human cells. Gpx3 has an oxidising effect. Trx1 has a reducing effect as thioredoxins catalyse the protein disulphide exchange of proteins through a conserved active site. The navy double-headed arrow indicates a potential communication between these two systems. Erv1, and its human homologue ALR, have an oxidising effect on Mia40/CHCHD4 and are responsible for re-oxidising the CPC motif of Mia40 as part of the oxidative folding process in the IMS. Cells with mutated/knocked down Erv1 have reduced levels of MIA substrate import.

Redox signalling is crucial, as it allows cells to rapidly adapt to a range of environmental stimuli. The cellular redox status is maintained by several antioxidant systems, primarily the thioredoxin (Trx) and glutaredoxin (Grx) systems. Redox signals produced by the essential functions of mitochondria such as oxidative phosphorylation (OXPHOS), fatty acid oxidation, and the synthesis of iron–sulfur clusters allow mitochondria to be integrated into the wider cellular context through the free diffusion of small molecules between the IMS and the cytosol via the semi-permeable OMM [58]. It is also important to note that the redox environment of the mitochondrial IMS is separately maintained from the cytosol and the matrix with each compartment being separately influenced by various factors [14]. This section of the review will focus on the protein modulators of Mia40 in the IMS including Gpx3, Hot13, Trx1, and their potential role(s) in regulating Mia40 functions (Figure 2).

### 4.1. Glutathione Peroxidase 3 (Gpx3)

Glutathione peroxidase 3 (Gpx3, also called Orp1 and Hyr1) is a thiol peroxidase and was one of the redox regulatory protein identified in the 2012 yeast IMS proteome study [10]. Gpx3 acts as a sensor for H_2_O_2_ within the cell and promotes the oxidation of the transcription factor Yap1 [59]. This leads to the formation of an intramolecular disulphide bond of Yap1 and its subsequent activation, occurring when there is an increased level of H_2_O_2_ that directly oxidises the Cys36 of Gpx3. Then, Gpx3 forms a transient intermolecular disulphide with Cys598 of Yap1, which is then oxidised and activated. Then, Yap1 translocates to the nucleus and induces the anti-oxidant response, which leads to the transcription of anti-oxidant response genes. This pathway is attenuated by thioredoxin, which reduces the disulphide in Gpx3 that is formed by an increase in H_2_O_2_ levels [59].

Gpx3 has a molecular weight (MW) of ≈26 kDa and undergoes alternative translation under H_2_O_2_ stress to form an 18-amino acid N-terminal (N18)-extended version of the protein that was identified as being targeted to the mitochondrial IMS [60,61]. This N-terminal 18 amino acid extension is encoded by a non-AUG codon and is capable of targeting other proteins to mitochondria both in vivo and in organello. Cells that lacked Gpx3 had aberrant mitochondrial morphology (displaying a “dumbbell” shape), along with decreased protein import and a lower IMM potential. These abnormal phenotypes could be reversed by expression of the mitochondria-targeted Gpx3. Note that Gpx3, which lacks the N18 extension, can also be imported into mitochondria with the import of both the extended and non-extended forms of Gpx3 being mostly unaffected by a lack of Mia40. This suggests that Gpx3 import is independent of Mia40 (an essential gene), and therefore, the protein has an unconventional IMS import mechanism. Furthermore, Gpx3 can interact with Mia40 to oxidise its reduced form, therefore maintaining its redox state as oxidised, with Gpx3 becoming rapidly reduced following incubation with Mia40 (Figure 2) [61]. Overall, these data suggest a mitochondria-specific role of Gpx3 in the IMS where it could interact with the IMS-localised Trx1 as the source of the reducing power for Gpx3 in this compartment, essentially mirroring the redox system of the cytosol.

### 4.2. Helper of Tim Protein 13 (Hot13)

Another modulator of Mia40 is the soluble IMS protein Hot13, which is a non-essential player of the MIA pathway, instead having an ancillary role. Hot13 is conserved among eukaryotes, contains 10 cysteines, and has a CHY zinc-finger domain, which provides the capacity to bind zinc [62]. Hot13 has been proposed to maintain Mia40 in a zinc-free state, thereby allowing for a more efficient re-oxidation by Erv1. Hot13 mutants have an increased sensitivity to the oxidising agent *t*-butyl hydroperoxide and impaired import of Mia40 substrates, leading to suggestions that Hot13 plays a role in IMS redox regulation. Additionally, Hot13 mutants, when compared to wild-type cells, have a greater level of reduced Mia40 but no difference in overall levels of Mia40 and Erv1. This leads to a proposed model where Hot13 acts as a metal chaperone to transfer zinc ions from Mia40 allowing for re-oxidation by Erv1 [63]. In conclusion, Hot13 has an important yet non-essential role in regulating the redox environment of the IMS and the process of oxidative folding.

### 4.3. Thioredoxin and Glutaredoxin

The thioredoxin system is comprised of thioredoxin (Trx), thioredoxin reductase (TrR), and nicotinamide adenine dinucleotide phosphate (NADPH) as an electron donor. *S. cerevisiae* contains three thioredoxins (Trx1, Trx2, and Trx3) and two thioredoxin reductases (Trr1 and Trr2) [64]. Trx3 and TrR2 are located in the mitochondrial matrix, while Trx1 and Trx2 are responsible for the cytosolic pathway. This system is crucial for regulating oxidative stress by providing electrons to the peroxiredoxins to remove ROS/RNS. Thioredoxins are small reductases (≈12 kDa) that catalyse disulphide exchange of proteins with a conserved CGPC motif in their active site. The characteristic thioredoxin fold is common between other proteins that modify thiol redox states such as glutaredoxins, and the basic structure of this fold is comprised of three alpha helices that surround four beta sheets with an active CX_2_C motif. The mechanism of the Trx system is similar to the Grx system with electrons being transferred by Trx to the substrate through thiol–disulphide exchange reactions [15].

Interestingly, Trx1 has previously been shown to directly interact with Mia40 in a large proteomic screen [65]. Trx1 along with Trr1 have been shown to dually localise in the IMS and the cytosol [10], raising the possibility that Trx1 is involved in influencing the IMS redox environment [61].

The process of disulphide bond formation is error-prone, and therefore, it seems logical that a reductase system would be present in this constricted sub-compartment in yeast mitochondria to allow for disulphide isomerisation as a means to achieve the correct disulphide bonds in IMS proteins. The reducing enzyme glutaredoxin 1 (GRX1) in the IMS was shown to modulate the oxidation of COX17 (a copper chaperone for the Cytochrome c Oxidase or COX complex), which is a mammalian Mia40 (CHCHD4) substrate [66]. Trx1 could conceivably have a similar role in yeast mitochondria, acting on Mia40 substrates to ensure the optimal functioning of the MIA pathway. This may not be required for all substrates but rather substrates with several cysteine residues, which have a more complex cysteine pair pattern. It has previously been suggested that this is logical, because the Grx and Trx reducing pathways can compensate for each other due to their overlapping functions, while the two are often found in the same compartments [55].

Further research is required to identify the interplay and communication of the mitochondrial-targeted Gpx3 and the levels of Trx1 in the IMS. It will be interesting to see how these two proteins act to influence the redox environment of this sub-compartment, especially as Erv1 has recently been identified to not be as active as previously thought [47]. A summary of the important proteins that are involved in redox regulation and signalling in the mitochondrial IMS as discussed in this section is given in Table 1.

## 5. Small Redox Molecules and Redox Homeostasis in the IMS

**Figure 3:**
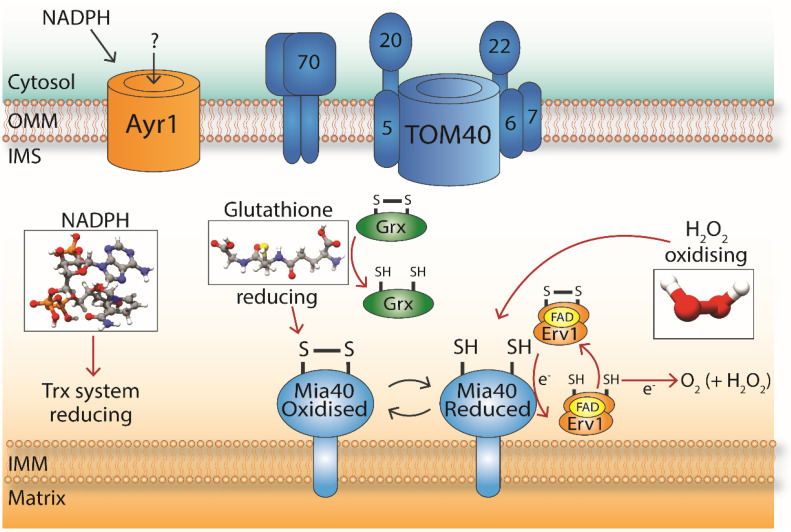
Small molecules affecting redox homeostasis in the IMS. Glutathione (GSH) has a reducing effect on Mia40/CHCHD4. H_2_O_2_ has an oxidising effect on Mia40/CHCHD4 and can easily cross mitochondrial membranes. NADPH has a reducing effect on Mia40/CHCHD4 and is utilised by the two main thiol-reducing pathways in the cell, the Trx and Grx pathways. Cytosolic NADPH has recently been implicated in the regulation of the newly discovered OMM channel protein Ayr1, whose function remains unknown. Small molecule structures were obtained from PubChem and visualised in Chimera version 1.15.0. PubChem CIDs are as follows; NADPH (5884), glutathione (124886) and H_2_O_2_ (784).

In addition to protein modulators of the redox environment in the IMS, there are also small molecules that play a role in redox regulation. These small molecules include hydrogen peroxide (H_2_O_2_) and glutathione (GSH), which act as signals and are important for initiating the redox response of the cell. H_2_O_2_ is classified as a reactive oxygen species (ROS), which are produced through normal cell metabolism and also include the superoxide anion (O_2_^−^) and the hydroxyl radical (OH^−^), which are all associated with oxidative stress [57]. ROS can act as signalling molecules by oxidising cysteine residues and forming disulphide bonds, which results in the alteration of a protein structure and therefore function. These disulphide bonds can be reversed by the disulphide reductases Trx and Grx, and this reversibility allows for the rapid alteration of protein function in response to ROS [57,67]. In the IMS, GSH has reducing activities and H_2_O_2_ has oxidising activities, and they both have a role in maintaining the redox environment of the IMS. In this section of the review, we will discuss potential dialogues between these molecules and the MIA pathway (Figure 3).

### 5.1. The Oxidant Hydrogen Peroxide (H_2_O_2_)

Reactive oxygen species are normally produced as by-products of cellular respiratory metabolism and can be harmful to cells when their concentrations are very high. Here, we focus on H_2_O_2_ in the IMS, its sites of production, and its influence on the redox state of the IMS: other ROS and RNS will be discussed briefly in subsequent sections. H_2_O_2_ is derived from the partial reduction of molecular oxygen and is more stable and less reactive than the ROS superoxide anion (O_2_^−^) because it does not contain unpaired electrons. Instead the reactivity of H_2_O_2_ is provided by its relatively weak O–O bond, which is susceptible to decomposition, resulting in the formation of a hydroxyl radical (OH^−^), which is very reactive [68,69].

H_2_O_2_ (and/or O_2_^−^) are produced by up to eleven distinct sites in mammalian mitochondria. These sites include processes such as the electron transport chain (ETC) and substrate oxidation, which have been covered extensively in a review elsewhere [70]. Generally, H_2_O_2_ is generated in places where there is a leakage of pairs of electrons, and there are various antioxidant defence systems in place to consume H_2_O_2_ and prevent damage to the cell. Additionally, H_2_O_2_ can easily pass through mitochondrial membranes, meaning that H_2_O_2_produced in the matrix can travel to the IMS through the IMM. This both increases the level of H_2_O_2_ in the IMS and may have a role in signalling to the cell through the buffering function of the IMS. H_2_O_2_ is generated in the IMS several ways. The copper–zinc isoform of Superoxide dismutase 1 (SOD1), which catalyses the rapid dismutation of two molecules of O_2_^−^ to produce H_2_O_2_ and O_2_, is present in the IMS. Superoxide anions are released to the IMS side of the IMM by various enzymes of the respiratory chain, notably complex III [5]. Additionally, the Erv1/ALR enzyme in its normal catalytic cycle also produces H_2_O_2_. During this process, Erv1 can pass the electrons directly to molecular oxygen with a concomitant production of H_2_O_2_. Daithankar et al. (Biochemistry 2009) first showed that Erv1 interacts with cytochrome *c* in vitro [44]. Allen et al. (J Mol Biol 2005) demonstrated that also in intact cells, cytochrome *c* (cyt *c*) is the in vivo oxidase for Erv1, as yeast cells mutated in cyt *c* cannot grow under anaerobic conditions. This demonstrated that Erv1 functionally links the Mia40-dependent import pathway to the Mia40-independent cyt *c* import pathway by transferring electrons from the incoming precursors to cyt *c* as an acceptor [41]. Later, Bihlmaier et al. in 2007 proposed that the interaction between Erv1 and cytochrome *c* may protect the cell from oxidative damage, as avoiding passing the electrons to molecular oxygen prevents H_2_O_2_ formation. It was concluded that this interaction allowed for electrons to be passed to the respiratory chain, which led to the production of water instead of H_2_O_2_ and simultaneously connected the ETC to the redox state of the IMS [42]. To remove H_2_O_2_ in the IMS, cytochrome *c* peroxidase (Ccp1) reduces the harmful H_2_O_2_ to water [43]. Additionally, the recently unconventionally targeted Gpx3 may also have a role in removing H_2_O_2_as thiol peroxidases are major antioxidants in cells and protect against H_2_O_2_ caused oxidative stress [61]. Both Ccp1 and Gpx3 can interact with components of the MIA pathway and are therefore capable of removing H_2_O_2_ from this process and the IMS [47].

In terms of signalling, H_2_O_2_ can directly oxidise select cysteine residues to induce signalling and is thought to be broadly non-specific. This oxidation of cysteine thiol groups can lead to structural changes in the protein, which can lead to a change of function. As mentioned above, Trx1 is capable of reducing these disulphide bonds with both Trx1 and Trr1 being identified in the IMS [10]. In the IMS, there is the potential for high local concentrations of H_2_O_2_ to manifest both from invaginations of the IMM and from the presence of respiratory chain enzymes [5]. Further study is required to determine which of the ≈51 IMS proteins in yeast and ≈127 in humans can be directly oxidised by H_2_O_2_ in mitochondria, and any effects of H_2_O_2_ diffusion through pores in the OMM may have on signalling between mitochondria and the cell. Interestingly, it has been reported that H_2_O_2_ signalling may be under the control of a signal transduction pathway, which links the ETC to the IMS-localised Syk pathway, with H_2_O_2_ inducing the phosphorylation of Syk. The authors suggest that this connection between the respiratory chain and the conserved Syk pathway produces a focused signalling response to the cell; however, only extracellular H_2_O_2_ was used in this study [68]. Nonetheless, it is important to appreciate the wider signalling aspect of H_2_O_2_ and not just the potential damage it can cause in cells.

The design of mitochondrial targeted reagents that are capable of independently inducing O_2_^−^ and H_2_O_2_ or disrupting mitochondrial thiol homeostasis (mitoParaquat and MitoChlorodinitrobenzoic acid respectively) will allow for further study into the effects of these process in the mitochondria [71]. Overall, H_2_O_2_ has an oxidising effect in the IMS, with signalling functions between the mitochondria and the wider cell. In the future, it will be interesting to look at any interplay between a potential reducing system in the IMS (Trx1 and Trr1) and H_2_O_2_ for maintaining the redox status of the IMS for optimal oxidative folding conditions (Figure 3).

### 5.2. The Reductant Glutathione (GSH)

GSH is a component of the glutaredoxin system, which contains glutaredoxins (Grx) and GSH reductases (GR). The glutaredoxin system is one of the major pathways that controls cellular redox homeostasis, in addition to the thioredoxin pathway. GSH has various key roles in reducing disulphide bonds, modulating iron–sulfur clusters biogenesis, and detoxifying ROS. Participating in these reactions frequently leads to the oxidation of GSH to GSSG, which contains a disulphide bond that links two molecules of GSH together, whilst the targets of GSH are left reduced. GSSG is reduced back to GSH by glutathione reductase (Glr) using electrons supplied by NADPH [45,72]. It is important to note that no GSH reductase has been found in the IMS. It has consequently been proposed that once GSH is oxidised to GSSG in this compartment, it is removed through porins and the TOM channel in the OMM. However, conclusive evidence supporting this GSH efflux mechanism from the IMS into the cytosol is still missing. GSH is synthesised in the cytosol and is imported into the mitochondria presumably through the TOM complex and porins in the OMM, which allow the free diffusion of small molecules. Additionally, there is a portion of this GSH that could be transported further to the matrix, likely through IMM carriers [45], although such specific IMM transporters have not yet been identified.

In cells, the majority of glutathione is in the reduced form GSH, and the ratio of GSH to GSSG (GSH:GSSG) is an indicator of oxidative stress, with a greater level of stress increasing the ratio of GSSG:GSH. This ratio varies throughout the cell, and understanding the local glutathione redox potential (E_GSH_) is key to further untangling the influence of redox processes. In 2012, Kojer et al. demonstrated that the GSH redox potential in the IMS is linked to the cytosol, with both compartments having a similar E_GSH_. They found that E_GSH_ in the IMS is determined by the glutathione reductase system in the cytosol with the two pools of GSH being kinetically connected. Furthermore, E_GSH_ in the matrix is regulated separately from the cytosol and the IMS. Interestingly, they reported that the local E_GSH_ has a role in maintaining the partially reduced redox state of Mia40, which is found to be around 70% oxidised. When Erv1 was depleted in cells, Mia40 was found to be increasingly reduced, which could be due to the GSH pool in this compartment [45]. However, the IMS can be further subdivided into the boundary IMS and cristae space, which could account for even further local variations in E_GSH_.

In the IMS, where disulphide bond formation takes place, GSH is responsible for recycling Grx back to its reduced active state, which can then reduce protein thiols. Note that the IMS contains the dithiol glutaredoxins Grx1 and Grx2, which catalyse S-glutathionylation, as well as Gpx3, Trx1, and Trr1 as mentioned above, but not its own glutathione-related enzymes [10,72]. Grx2 has been identified as the main Grx in the IMS of yeast mitochondria and was proposed to aid oxidative folding, despite the reducing GSH pool [73]. The process of S-glutathionylation involves a covalent disulphide bond between a molecule of GSH and the cysteine residue of a protein. Through this post-translational modification, cysteine residues in IMS proteins could be modified by ROS and RNS, with implications for redox signalling. Recently, it has been proposed that human Mia40 (CHCHD4) undergoes glutathionylation, which is then capable of transferring electrons directly to cytochrome *c* [74]. These authors suggest that glutathionylation of human Mia40 occurs to control the levels of ROS levels normally produced by electron transfer complexes III and IV. Additionally, GSH in the IMS has been proposed to be an important factor for the efficiency of the oxidative folding pathway by (i) increasing the speed of import into this sub-compartment [73] and (ii) providing a proofreading function within the disulphide relay process [75]. However, in vivo data for such a function in the IMS are still lacking. We should note that although GSH plays important roles in redox homeostasis in the IMS (Figure 3) and the rest of the cell, an alternative model has been proposed in which the main role of GSH is to act as a key modulator of iron metabolism, while modulating the thiol state would be an ancillary role [76].

### 5.3. NADH/NADPH

Nicotinamide adenine dinucleotide (NAD^+^), reduced Nicotinamide adenine dinucleotide (NADH), nicotinamide adenine dinucleotide phosphate (NADP^+^), and reduced nicotinamide adenine dinucleotide phosphate (NADPH) are now well established as important enzyme cofactors and signalling mediators of fundamental metabolic and biosynthetic processes and antioxidant functions in the cell [77]. Recent studies in living cells, performed by means of genetically modified cells and compartment-specific fluorescent probes, have further confirmed the spatio-temporal control of NADH and NADPH homeostasis in specific subcellular compartments such as the nucleus, cytoplasm, and mitochondria, thereby highlighting the importance of these molecules in compartment-specific local metabolic processes [77]. In this section of the review, we will discuss potential connections between NADH/NADPH-regulated processes and the redox-regulated MIA pathway in the mitochondrial IMS (Figure 3).

NADPH, which functions as a cofactor and electron donor, is implicated in energy metabolism, biosynthetic pathways, and in the regeneration of the antioxidant capacity of the cell. NADPH-dependent redox systems are controlled by pools of NADPH produced mainly in the cytoplasm and mitochondrial matrix [77]. Published reports point toward a role for cytosol-generated NADPH and NADPH-dependent Glr1 in the regulation of the pool of GSH that is transported to the IMS via porins’ activity in the OMM of mitochondria [45]. As the cytosol-dependent GSH redox potential influences the redox state of the IMS-localised Mia40 [45], it will be interesting in the future to elucidate further links between the potential reducing systems in the IMS and the cellular redox state. This is particularly relevant, since the Trx system (which has been found to dually localise in the cytosol and the IMS) also operates under the control of NADPH.

Reports indicate that NADPH may control the transport of other, yet unknown small molecules and/or metabolites via OMM channels such as the newly discovered, NADPH-regulated, 1-acyldi-hydroxyacetone-phosphate reductase (Ayr1). The function of Ary1 remains unknown, including what the channel is responsible for transporting. The presence of NADPH was reported to stimulate the frequency of channel gating between different states, and in the absence of NADPH, the potential of the channel was reversed [78]. Therefore, there may be regulated traffic in and out of the IMS that is under the control of cytosolic NADPH and hence redox state control.

In human cells, an NAD(P)H-dependent redox process was also found to influence the interaction of the human homolog of Mia40 (CHCHD4) with its regulatory partner AIF [52]. The flavoprotein AIF, which carries a central NAD^+^/NADH binding segment, exists both in monomer and dimer forms in the mitochondrion [79,80,81,82,83,84,85,86,87]. Upon the interaction of AIF with NADH or NADPH, the monomer/dimer equilibrium shifts towards the dimer. The interaction of NAD(P)H with AIF triggers the formation of dimeric and stable FADH-NAD(P) charge-transfer complexes (CTC) that are not efficient in electron transfer [82,83,84,85,86,87]. Hangen et al. in 2015 showed that the conformational state of AIF, which is promoted by the binding of reduced pyrimidine nucleotides (NADH or NADPH), favors its interaction with CHCHD4 [52]. Although it is not yet clear how the NAD(P)H-driven conformational change of AIF controls the availability and function of CHCHD4 in the IMS, the existence of a human mitochondriopathy-associated pathogenic mutation of AIF (G308E), which is located within its NAD^+^/NADH-binding domain and impairs the interaction with CHCHD4, is an indicator of the relevance of NADH/NADPH homeostasis in the physiological functioning of the MIA import pathway [52,88].

A summary of the important small redox-active molecules associated with redox regulation in the mitochondrial IMS discussed in this section is given in Table 2.

## 6. Interplay of Proteins and Small Molecules in Fe-S Cluster Biogenesis and Calcium Signalling in the IMS

**Figure 4:**
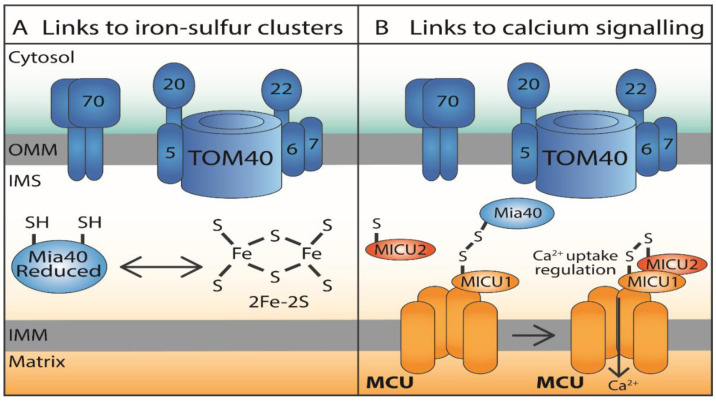
Potential signalling mechanisms linked to the MIA pathway. (**A**) Links between Mia40/CHCHD4 and iron–sulfur clusters (ISCs). Mia40/CHCHD4 has been previously reported to bind a rhombic 2Fe-2S cluster in a dimer form and to play a role in the export of ISCs. However, the role of IMS and in particular redox-regulated Mia40 in ISCs biogenesis remains to be fully detailed. (**B**) Links between Mia40/CHCHD4 and calcium signalling. Mia40 has been reported to be responsible for introducing an intermolecular disulphide bond, which links MICU1 to MICU2. This mixed disulphide between Mia40 and MICU1 was found only when mitochondrial membrane potential was intact. Overall, this interaction with Mia40 links the regulation of calcium uptake by mitochondria to the redox-regulated folding machinery in the IMS.

As the IMS is a constricted compartment, there is likely to be some communication between the different proteins involved in the IMS machinery and other IMS-related pathways, particularly signalling pathways. It has previously been suggested that there is crosstalk between the mitochondrial GSH and Trx systems in the matrix, which does not occur in the cytosol [72]. We propose that a similar crosstalk may also occur in the IMS to maintain the redox state of this sub-compartment. This section will discuss the potential links of the IMS-localised and redox-regulated MIA pathway to (i) iron-sulfur clusters and (ii) calcium signalling, highlighting both the small molecules and the proteins that could be involved (Figure 4).

### 6.1. Iron–Sulfur Clusters

Iron–sulfur clusters (ISCs) are cofactors, specifically metal prosthetic groups that are involved in a wide range of cellular pathways such as protein translation, energy production, and DNA replication and repair [89,90]. The simplest ISC is the rhombic 2Fe-2S, which can be found in ETC complexes I and II, whilst duplication of the rhombic form results in the cubic 4Fe-4S form. ISCs are highly reactive, which is a characteristic that makes them suitable for a range of catalytic reactions, and susceptible to oxidation, which leads to their inactivation [90,91,92]. There are three identified assembly pathways of ISCs: two are present in the mitochondria and one is present in the cytosol. Both the ISC assembly system and the iron–sulfur exporter (ISE) system are present in the mitochondria, while the cytosolic iron–sulfur assembly system (CIA) is present in the cytosol [1,91]. There are potential links between IMS-localised Mia40-dependent machinery and ISCs (Figure 4), which first emerged in 2013 when Mia40 was found to bind a 2Fe-2S cluster in a dimer form. The ISC bound to reduced Mia40 was proposed to be coordinated by the cysteine residues of the redox-active CPC motif of two Mia40 molecules, and the addition of H_2_O_2_ (which oxidises Mia40) leads to the disassembly of the ISC [93]. Additionally, the authors raised questions on whether changes in the two populations of Mia40 in the IMS, one oxidised (not bound to iron) and the other reduced (apo ISC bound), have a role in responding to external stimuli, as high levels of H_2_O_2_ lead to ISC dissociation [93]. Interestingly, human Mia40 (CHCHD4) has also been suggested to play a role in the export of ISCs, with depletion of CHCHD4 resulting in the accumulation of iron in mitochondria, along with a decrease in the activity and stability of cytosolic Fe-S containing enzymes. It was speculated that since the CPC motif of Mia40 is required for both protein import and ISC binding, the redox environment of the IMS could have an effect on influencing the function of Mia40 either in protein import or in the export of ISC [89]. However, overall, the role of Mia40 in regulating cellular iron homeostasis is still disputed, and the exact role of redox-regulated Mia40 in the biogenesis of ISCs remains to be fully dissected. It is important to consider that a population of Mia40 must be kept reduced (potentially by Trx1 or GSH) to allow interaction with ISCs and perhaps facilitate ISC export.

### 6.2. Calcium Signalling

Calcium uptake into mitochondria of higher eukaryotes is mediated by the mitochondrial calcium uniporter (MCU), which is an IMM-anchored calcium-conducting protein that functions in concert with regulatory proteins MICU1 (Mitochondrial calcium uptake 1) and MICU2 (Mitochondrial calcium uptake 2). The MCU-mediated uptake of calcium is driven by the membrane potential of the IMM [94]. During evolution, some species of yeast including *S. cerevisiae* have lost MCU and instead contain MCU homologs [95]. Tight control of calcium uptake by mitochondria is essential due to the critical role of calcium in cell death, autophagy, and activating three Krebs cycle dehydrogenases, which in turn affect ATP production via OXPHOS. Other components of the human MCU include the MCU paralog MCUb, which has 50% similarity to MCU and a similar structure differing slightly in a loop region, and EMRE, which is a small IM 10 kDa protein [96]. Recently, two homologs of the scaffold factor of MCU (MCUR1) were identified in S. cerevisiae, namely Put6 and Put7 but with a role in proline metabolism [97]. Interestingly, MICU1, which is one of the regulator partners of MCU and therefore calcium uptake, resides in the IMS, which may explain how mitochondria respond to calcium levels in the cytosol [12]. MICU1 regulates MCU by keeping the pore closed when there is a low level of calcium, and it mediates the pore opening when higher calcium concentrations are available to facilitate calcium transport across the IMM. A Mia40 interactome study identified MICU1 as a Mia40 interactor, therefore linking disulphide bond formation to calcium signalling [98]. MICU1 contains a matrix-targeting signal of 33 residues, making its import independent of Mia40 and instead dependent on the membrane potential of the IMM. Mia40 was reported to be responsible for introducing an intermolecular disulphide bond, which promotes the heterodimerisation of MICU1 to MICU2: MICU2 inhibits MICU1 and does not interact with Mia40. When this disulphide bond is introduced to MICU1 by Mia40, association of the MICU1–MICU2 heterodimer with MCU is dependent on IMS calcium levels. Association occurs when there is a low level of calcium present, and dissociation occurs when there is a high level of calcium. It is noteworthy that the disulphide between Mia40 and MICU1 was found only when the membrane potential was intact [98]. Overall, this interaction with Mia40 provides a link between the regulation of calcium uptake by the mitochondria and the redox-regulated oxidative folding machinery in the IMS, as regulators of mitochondrial calcium signalling (MICU1 and MICU2) are resident in the IMS (Figure 4).

## 7. Conclusions and Future Outlook

In this review, we have highlighted some critical aspects of how redox regulation and signalling converge in the mitochondrial intermembrane space at the MIA pathway. Optimal operation of these processes requires a finely tuned interplay between proteins and small molecules. Despite several important advances in identifying the important players and in elucidating some of the key mechanisms, our understanding is not yet complete. Several proteins components that we have discussed in this review have been identified as regulators or potential interactors of the disulphide relay machinery, but it is not clear whether they operate in a constitutive manner or they are part of mechanisms activated in response to metabolic changes or stress conditions. Furthermore, small molecules and ROS, which can also influence the function of the MIA machinery need to be kept under balance and detoxified to avoid deleterious effects and keep them within a concentration regime that is beneficial for signalling and not detrimental. Furthermore, the influence of the most damaging ROS generated in mitochondria (the super oxide anion), which has at least eight sites of production [99], is unclear and will be the subject of future studies. For example, additional effects of ROS on lipid peroxidation, which can cause membrane damage and lead to further ROS production, are very poorly understood.

The critical link of the MIA machinery to generalised mitochondrial dysfunction and human disease is exemplified by at least three different axes. First, the mutation or abnormal expression of components of the MIA machinery and its interactors such as AIF are involved in several human diseases [26,55]. Second, substrates of the MIA pathway are linked also to primary mitochondrial diseases (such as several mitochondriopathies related to COX deficiencies) and to more common neurodegenerative diseases (such as for example CHCHD2 and CHCHD10, which are linked to amyotrophic lateral sclerosis) [100]. Finally, CHCHD4 has recently been found to be required for PINK1 stabilisation in the absence of mitochondrial membrane potential, whilst the inhibition of CHCHD4 by antioxidants such as GSH could suppress PINK1 accumulation [101]. This opens up the possibility that MIA machinery may have unknown links to PINK1-dependent mitophagy and potentially affect not just mitochondria biogenesis but more broadly mitochondria quality control [102]. This represents an interesting example of two distinct types of signalling cues: redox-signalling and phosphorylation-signalling converging at the mitochondria via the MIA system. In broader terms, fully teasing out the molecular links between different types of regulation and signalling as they coalesce at the intermembrane space could advance our understanding of mechanisms that maintain mitochondrial fitness and could help the development of potential therapeutic targets in human diseases relating to mitochondria dysfunction.

## Figures and Tables

**Table 1:** Important proteins involved in redox regulation and signalling in the mitochondrial IMS discussed in this review.

Protein Name	Function	References
Mia40/CHCHD4	Oxidoreductase donates a disulphide bond from its CPC motif to the substrate proteins, thereby catalysing their oxidative folding. Mia40/CHCHD4 requires re-oxidation to undergo another round of oxidative folding.	[10,17,18,19,20,21,22,23,25,26,27,28,29,30,31,49,50,51,52,53,54,55,56]
Erv1/ALR	FAD-dependent sulfhydryl oxidase, responsible for re-oxidising Mia40/CHCHD4 to allow another cycle of disulphide bond formation on the protein substrates. Electrons from the reduced Mia40/CHCHD4 CPC motif flow to the N-terminal shuttle CX2C motif of Erv1/ALR, following a “substrate mimicry” mechanism.	[20,24,31,32,33,34,35,36,37,38,39,40,41,42,43,44,45,46,47,52]
Gpx3 (also called Orp1 and Hyr1)	A thiol peroxidase acting as an H_2_O_2_ sensor and promotes the oxidation of the transcription factor Yap1 in the cytosol, inducing the anti-oxidant response. Gpx3 undergoes alternative translation under H_2_O_2_ stress, forming an N-terminally extended version that is targeted to the IMS. Gpx3 can reoxidise reduced Mia40.	[10,59,60,61]
Hot13	Hot13 is conserved amongst eukaryotes, but it is a non-essential protein. It is associated with the MIA pathway as it allows a more efficient re-oxidation of Mia40 by Erv1, potentially by maintaining Mia40 in a zinc-free state.	[62,63]
Thioredoxin	Thioredoxins are ubiquitous proteins with a major role in regulating oxidative stress by providing electrons for the removal of ROS/RNS and reducing disulphide bonds. Trx1 is dually localised between the cytosol and the IMS. It interacts with Mia40, and it may influence the redox environment of the IMS.	[10,15,61,64,65]
Glutaredoxin	Glutaredoxin has a similar mechanism to thioredoxin, transferring electrons to the substrate proteins through thiol–disulphide exchange reactions. Grx1 is also a reducing enzyme and modulates the oxidation of COX17, which is a Mia40 substrate.	[15,55,66]

**Table 2:** Small redox-active molecules associated with redox regulation in the mitochondrial IMS discussed in this review.

Molecule Name	Function	References
Hydrogen peroxide (H_2_O_2_)	H_2_O_2_ is a small molecule oxidant and can easily pass through the mitochondrial membranes. H_2_O_2_ is produced by Erv1/ALR when electrons are passed to molecular oxygen as part of the cycle to re-oxidise Mia40. Additionally, H_2_O_2_ can directly oxidise select cysteine residues to induce signalling through structural changes in the protein. Trx1 is usually the enzyme that reduces disulphide bonds created by H_2_O_2_.	[5,10,41,42,43,44,47,61,68,69,70,71]
Glutathione (GSH)	A component of the glutaredoxin system, GSH has various roles in the reduction of disulphide bonds. The majority of GSH in cells is in the reduced form of GSH. The ratio of reduced/oxidised GSH is a measure of oxidative stress. The GSH redox potential in the IMS is linked to the cytosol, and the two pools of GSH are kinetically connected. GSH in the IMS has been proposed to increase the rate of protein import and provide a proofreading function for the disulphide relay process.	[10,45,72,73,74,75,76]
NADH/ NADPH	NADPH is an important cofactor and electron donor, influencing both the glutaredoxin and thioredoxin systems and may have a critical role in redox homeostasis in the IMS. NAD(P)H-dependent redox control influences the interaction of CHCHD4 with AIF via dimerisation of AIF in human cells.	[45,52,77,78,79,80,81,82,83,84,85,86,87,88]
